# Quantitative photothermal analysis and multispectral imaging of dental structures: insights into optical and thermal properties of carious and healthy teeth

**DOI:** 10.1117/1.JBO.29.1.015003

**Published:** 2024-01-27

**Authors:** Elnaz Baradaran Shokouhi, Damber Thapa, Robert Welch, Koneswaran Sivagurunathan, Andreas Mandelis

**Affiliations:** a University of Toronto, Center for Advanced Diffusion-Wave and Photoacoustic Technologies, Department of Mechanical and Industrial Engineering, Toronto, Ontario, Canada; b University of Toronto, Institute for Advanced Non-Destructive and Non-Invasive Diagnostic Technologies, Toronto, Ontario, Canada

**Keywords:** dental caries, pulse photothermal radiometry, multispectral imaging, layered turbid tissue, micro-computed tomography

## Abstract

**Significance:**

In the analysis of two-layered turbid dental tissues, the outer finite-thickness layer is modeled by an optical transport coefficient distinct from its underlying semi-infinite substrate layer. The optical and thermophysical parameters of healthy and carious teeth across the various wavelengths were measured leading to the determination of the degree of reliability of each of the fitted parameters, with most reliable being thermal diffusivity and conductivity, enamel thickness, and optical transport coefficient of the enamel layer. Quantitative pixel-by-pixel images of the key reliable optical and thermophysical parameters were constructed.

**Aim:**

We introduced a theoretical model of pulsed photothermal radiometry based on conduction-radiation theory and applied to quantitative photothermal detection and imaging of biomaterials. The theoretical model integrates a combination of inverse Fourier transformation techniques, avoiding the conventional cumbersome analytical Laplace transform method.

**Approach:**

Two dental samples were selected for analysis: the first sample featured controlled, artificially induced early caries on a healthy tooth surface, while the second sample exhibited natural defects along with an internal filling. Using an Nd:YAG laser and specific optical parametric oscillator (OPO) wavelengths (675, 700, 750, and 808 nm), photothermal transient signals were captured from different points on these teeth and analyzed as a function of OPO wavelength. Measurements were also performed with an 808-nm laser diode for comparison with the same OPO wavelength excitation, particularly for the second sample with natural defects.

**Results:**

The findings demonstrated that the photothermal transient signals exhibit a fast-decaying pattern at shorter wavelengths due to their higher scattering nature, while increased scattering and absorption in the carious regions masked conductive and radiative contributions from the underlayer. These observations were cross-validated using micro-computed tomography, which also enabled the examination of signal patterns at different tooth locations.

**Conclusions:**

The results of our study showed the impact of optical and thermal characteristics of two-layered turbid dental tissues via an inverse Fourier technique, as well as the interactions between these layers, on the patterns observed in depth profiles.

## 1 Introduction

In recent decades, there has been a growing trend in utilizing photothermal techniques for nondestructive evaluation of thermal and optical properties of biological samples. These techniques are particularly focused on accurate measurements of the optical scattering and absorption properties of both normal and abnormal tissue. Pulsed photothermal radiometry (PPTR) relies on the thermal infrared emission of a material response to a laser pulse, followed by heat transport through radiation and conduction.^1^ The PPTR technique conveys information about subsurface features in the form of temperature depth integrals. A key aspect of this technique is to identify early defects and subsurface characteristics and to assess the depth of such defects through heat pulse characteristics and transient changes in temperature distribution over time. Typically, when dealing with such scenarios, a homogeneous tissue model with a single layer of infinite depth is used, and the diffusion equation is solved at the surface while considering relevant boundary conditions. Nevertheless, biological tissues, such as skin or teeth, are often not uniform in their composition and should be considered as layered structures. Hence, PPTR was used to study layered materials and more specifically the effect of a subsurface absorber on the time-dependence of the PPTR signal such as human skin. Initially, a simple theoretical model was developed to understand the PPTR signal transient.^2^ The model assumed thermally homogeneous layers with the semi-infinite substrate having a finite absorption coefficient. Although the uppermost layer’s absorption coefficient was set to zero, the obtained PPTR signal displayed a distinctive rounded peak. This peak suggested the existence of an absorptive material beneath the surface at a specific depth. Another study introduced a diffusion model that explained the photon flux and considered the influence of scattering and absorption through the epidermal, dermal, and subcutaneous tissue layers of skin, including explicit treatment of anisotropic scattering and diffuse specular reflection.^3^ Later, a theory was derived based on the diffusion approximation of the radiative transport equation which accurately models the time course of the detected PPTR signal in semi-infinite scattering and absorbing one-layer media.^4^ The model was improved further to describe the PPTR signal based on optical diffusion theory for a two-layer, semi-infinite medium with a surface layer having different optical absorption and scattering properties than the underlying layer while the thermal properties of both layers remained constant.^5^

Teeth are complex multilayered biological tissues that are made up of different tissues, including enamel, dentin, cementum, and pulp. Dental caries is a disease that involves the gradual breakdown of hard tissues in teeth, starting with the enamel and later progressing to the dentin.^6^ This decay occurs in small spots and is caused by the acids produced by bacteria when they break down carbohydrates from food. Early enamel lesions generally exhibit an intact hard outer surface, while subsurface demineralization takes place.^7^ Despite this, the tooth surface is not disrupted due to the preferential remineralization that occurs there, which is facilitated by higher concentrations of calcium and phosphate ions. Pores in the lesion due to partial dissolution of mineral crystals scatter visible and near-IR light strongly. This porosity caused by selective dissolution increases the scattering coefficient in the lesion by one to two orders of magnitude compared to healthy tissue.^8^ Light scattering in sound enamel and dentin is sufficiently strong in the visible range to obscure transmission through the tooth. Enamel only weakly absorbs visible light and scatters it less as the wavelength increases, especially in the near-infrared region.^9^ Several theoretical models utilizing photothermal radiometry have been established for the quantitative assessment of the optical and thermal characteristics of both healthy and demineralized dental enamel. The optical and thermal properties of teeth have been the focus of a number of studies. Zuerlein et al.^10^ used a simple one-dimensional heat conduction equation to determine the absorption depth of bovine enamel at 9.6, 10.3, and 10.6  μm wavelengths. In that study, the absorption depths at various wavelengths and other parameters such as pulse duration and the number of incident pulses were required to precisely control the treatment depth of dental enamel. Nicolaides et al.^11^ used a coupled diffuse-photon-density and thermal-wave technique for quantitative dental measurements using simultaneous frequency-domain PTR and luminescence. The technique works by measuring the thermal and luminescent responses of teeth to a modulated laser beam, and using this information it determines the thermal diffusivity and luminescence properties of the dental tissue.^11^ Later, the theoretical model that was originally developed for frequency-domain photothermal radiometry of single layer dental tissue (enamel or dentin) was further improved to include multiple layers and was extensively used to study the optical and thermal characteristics of both healthy and demineralized enamel at a specific excitation wavelength of 659 nm.^12^

In this paper, a one-dimensional PPTR theoretical model is constructed to describe concurrent conduction and radiation heat transfer within a two-layer substance, with particular focus on dental materials and structure. With the intention of investigating the thermal and optical characteristics of healthy and demineralized enamel, we consider the problem of an absorbing and scattering demineralized enamel layer in intimate contact with a semi-infinite absorbing and scattering healthy enamel substrate where each layer has its unique photothermal properties. In addition, on the surface of the demineralized enamel, there is a mineralized superficial enamel layer overlying the intact surface layer and body of the demineralization lesion, responsible for localized light absorption and scattering, also verified by transverse microradiography as the gold standard to obtain the mineral profile of carious and healthy enamel, particularly the depth of carious lesions.^13^^–^^15^ Regarding conduction and radiation heat transfer within sound enamel, the finite upper layer comprises enamel, while the underlying layer consists of semi-infinite dentin. Here, we use the PPTR Fourier spectral theory to evaluate the various system parameters in the frequency domain where a complete analytical expression can be derived, then linked to the time-domain theory and experimental data through an efficient inverse Fourier transformation algorithm, thereby using a hybrid analytical approach so that the theoretical interpretation of experimental signals can adequately take into account all thermal and optical parameters of the system under examination in a rigorous combined conduction-radiation heat transfer problem. A similar technique was introduced in an earlier study, where a controlled experimental approach was employed. This involved utilizing solid specimens, such as black rubber and anodized aluminum, as a strongly absorbing second layer with well-defined optothermal characteristics.^16^ An essential difference between this study and prior research lies in the utilization of biological samples such as teeth that contain both absorption and scattering coefficients in all layers, which allows the quantitative examination and evaluation of how the optical and thermal properties of healthy and demineralized enamel vary depending on the wavelength using an Nd:YAG pulsed laser which pumps an optical parametric oscillator (OPO) for wavelength tunability.

## 2 Theoretical Model

Following optical-to-thermal energy conversion in a tooth absorbing optical energy, the produced heat contributes to the photothermal signal in two ways: one way is conductive, originating from a proximity to the surface, controlled by the enamel thermal diffusion length and thermal diffusivity. The other is radiative, involving infrared emission from deeper areas accessed by the absorbed and scattered laser-induced optical field. Initially, in the time-domain, the conductive heat pattern emerges close to the surface within a time-dependent thermal diffusion length. This pattern ultimately reaches the surface, adding to the radiative infrared emission signal. Direct thermal infrared emission takes place from all absorption sites, with infrared photons passing through the enamel due to its mid-infrared transmittance windows. Light can become trapped in highly light scattering areas with poor thermal properties, such as demineralized regions. As a result, the thermal-wave distribution generated thereafter moves closer to the surface, compared to a healthy region.

**Fig. 1:**
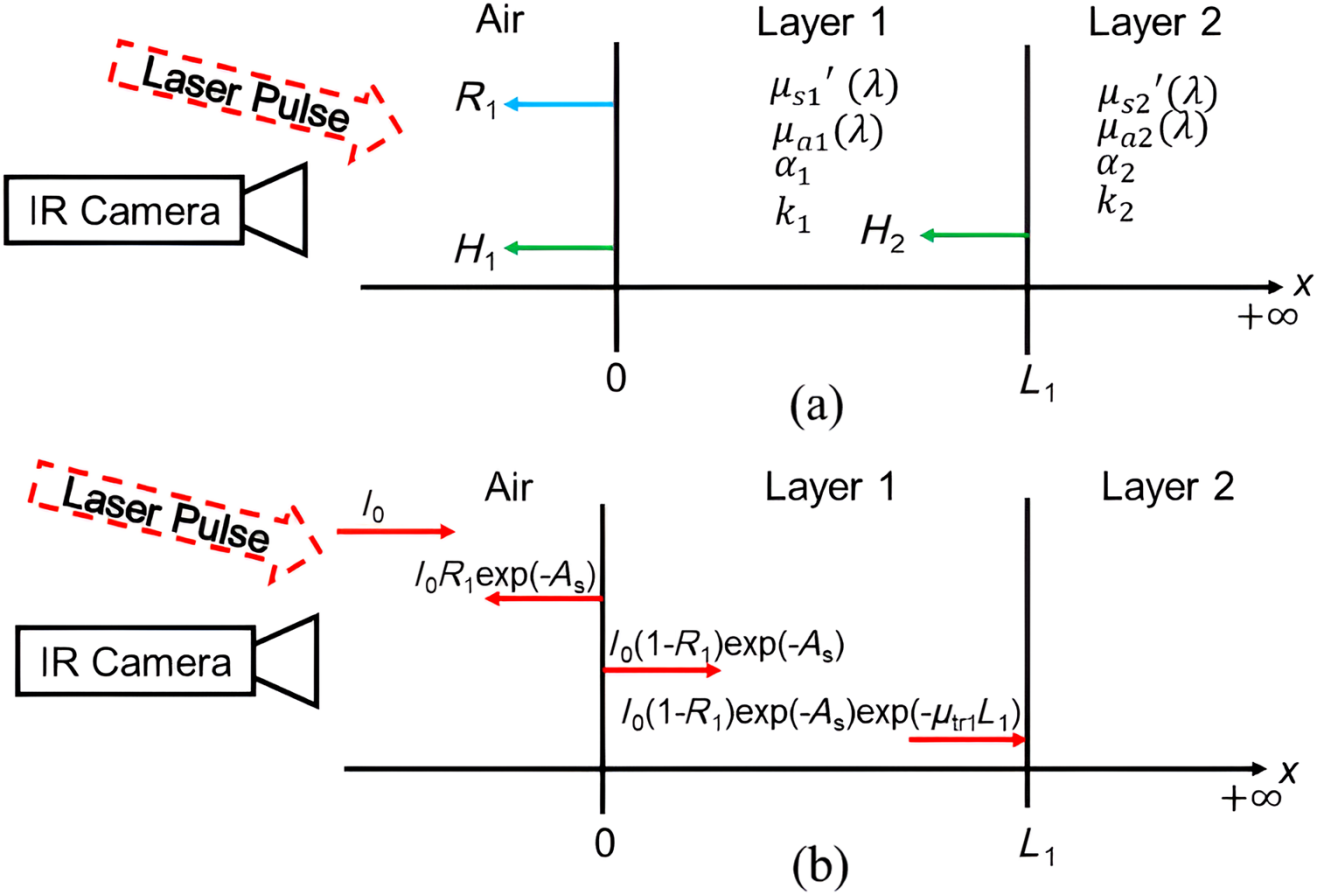
(a) Schematic geometry of the layers of demineralized enamel analyzed in this work. (b) Inter-reflection scheme inside the upper tooth layer formed by a demineralizing agent.

A one-dimensional schematic for geometry of the tooth structure is shown in Fig. 1(a). At x=0, the tooth is uniformly irradiated by an nanosecond optical pulse of the Dirac δ(t) type. Layer 1, of thickness $L_{1}$, has an optical absorption coefficient $\mu_{a1}$, and reduced scattering coefficient $\mu_{s1}^{\prime }$, and is in intimate contact with a semi-infinite layer 2 at $x=L_{1}$ with optical absorption coefficient $\mu_{a2}$ and reduced scattering coefficient $\mu_{s2}^{\prime }$. For PPTR analysis of healthy teeth, layers 1 and 2 represent healthy enamel and dentin, respectively. Conversely, for demineralized teeth, layer 1 is the demineralized enamel that comes in contact with a sound enamel back layer. In addition, for demineralized teeth, there exists a thin intact surface layer at x=0 with thickness $x=L_{0}$. At the excitation wavelength, this layer has absorbance, $\mu_{a0}$, and scattering, $\mu_{s0}^{\prime }$. The internal inter-reflection, $R_{1}$, effect is taken into account only in the demineralized layer and the effect of the inter-reflections between boundaries of the demineralized tooth is shown in Fig. 1(b).

The photothermal theory is developed in the frequency domain as the spectrum of the PPTR response and is later converted into the time domain (response to pulsed laser excitation) through a numerical inverse Fourier transform method. The time-domain temperature field generated after the onset of the incident pulse is the signal of interest in this work.

The frequency-domain photothermal equations in layers 1 and 2 and the surrounding gas (air) volume are

$$\frac{d^{2}}{dx^{2}}T_{g}(x,\omega )-(\frac{i\omega }{a_{g}})T_{g}(x,\omega )=0;\;x\leq 0,$$ (1a)

$$\frac{d^{2}}{dx^{2}}T_{1}(x,\omega )-(\frac{i\omega }{a_{1j}})T_{1}(x,\omega )=-(\frac{\mu_{a1}}{2k_{1}})I_{1};\;0\leq \;x\leq L_{1,j},$$ (1b)

$$\frac{d^{2}}{dx^{2}}T_{2}(x,\omega )-(\frac{i\omega }{a_{2}})T_{2}(x,\omega )=-(\frac{\mu_{a2}}{2k_{2}})I_{2};\;x\geq L_{1,j},$$ (1c)

The following parameters are defined: ω, an arbitrary angular frequency of the Fourier spectrum of $T_{g}(x,t)$, as well as $T_{1}(x,t)$ and $T_{2}(x,t)$ which represent the temperature field of the air layer, the sample first layer, and the second layer, respectively. The symbols $\alpha_{g}$, $\alpha_{1}$, and $\alpha_{2}$ signify the thermal diffusivity ($m^{2}/s$) of air, layer 1, and layer 2, respectively, while $k_{j}$ represents the associated thermal conductivity (W/m·K).

Light entering the tooth is subject to scattering and absorption. A collimated laser beam normal to the surface has a small portion of the light reflected at the surface of the enamel lesion at x=0, and at the second layer interface, and the remaining light is attenuated in the tissue by absorption and scattering according to the Beer–Lambert law:

$$I_{1}(x)=I_{0}(1-R_{1})\exp(-A_{s})\exp(-\mu_{t1}x);\;0\leq x\leq L_{1},$$ (2a)

$$I_{2}(x)=I_{0}(1-R_{1})\exp(-A_{s})\exp(-\mu_{t1}L_{1})\exp(-\mu_{t2}(x-L_{1}));\;x\geq L_{1},$$ (2b)

where $\mu_{t}=\mu_{a}+{\mu_{s}}^{\prime }$ is the optical transport coefficient ($m^{-1}$) pertaining to each layer, $I_{0}$ is the light source intensity incident at the surface ($W/m^{2}$), $A_{s}$ is the optical absorbance of the thin layer ($=\mu_{t0}$ × thickness $L_{0}$), with $A_{s}=\underset{L_{0}\to 0}{\lim}(\mu_{t0}L_{0})$. For theoretical analysis of healthy tooth, $A_{s\;}=0$ and $R_{1}$ is not taken into account. Equations 1(a)–1(c) are solved subject to temperature and heat flux continuity at all interfaces:

$$T_{g}(0)=T_{1}(0),$$ (3a)

$$k_{g}{\frac{\partial T_{g}}{\partial x}|}_{x=0}={k_{1}\frac{\partial T_{1}}{\partial x}|}_{x=0}-H_{1}T_{1}(0)+I_{0}\;\exp(-A_{s}),$$ (3b)

$$T_{1}(L_{1})=T_{2}(L_{1}),$$ (3c)

$$k_{1}{\frac{\partial T_{1}}{\partial x}|}_{x=L_{1}}=k_{2}{\frac{\partial T_{2}}{\partial x}|}_{x=L_{1}}+I_{0}(1-\exp(-A_{s}))\exp(-\mu_{t1}L_{1}),$$ (3d)

which yield the following expressions for the temperature distributions in the three regions:

$$T_{g}(x,\omega )=C_{1}\;\exp(\sigma_{g}x),$$ (4a)

$$T_{1}(x,\omega )=(\frac{\mu_{a1}}{2k_{1}(\sigma_{1}^{2}-\mu_{a1}^{2})})I_{1}+C_{2}\;\exp(\sigma_{1}x)+C_{3}\;\exp(-\sigma_{1}x),$$ (4b)

$$T_{2}(x,\omega )=(\frac{\mu_{a2}}{2k_{2}(\sigma_{2}^{2}-\mu_{a2}^{2})})I_{2}+C_{4}\;\exp(-\sigma_{2}x),\;$$ (4c)

where $C_{1}$, $C_{2}$, $C_{3}$, and $C_{4}$ are integration constants determined by the boundary conditions. $\sigma_{j}=(1+i){\left(\omega /2\alpha_{j}\right)}^{1/2}$ has the units of $m^{-1}$, and the physical meaning of a dispersive complex wave-number element. To complete the theoretical description, radiation heat transfer was taken into account at every surface of the system for both healthy and demineralized layers of the tooth. The coefficient $H_{1}$ (${W\cdot m}^{-2}{\cdot K}^{-1}$) is a radiation heat transfer coefficient which represents the radiative flux in an outward direction at x=0 that is incident on the IR detector. At the interface located at $x=L_{1}$, $H_{2}$ takes into account the heat transfer caused by radiation emitted from the front surface of layer 2. The thermal energy emitted at the interface between layer 2 and layer 1 can be reabsorbed by the first layer if the latter has a high ability to absorb and emit infrared radiation. Solving the boundary-value problem with the system of three equations in regions g, 1, and 2 and the boundary conditions, gives the expression for the spectrally averaged PPTR detected signal:

S^(ω)=μ¯IR[∫0L1T1(x,ω)exp(−μ¯IRx)dx+∫L1∞T2(x,ω)exp(−μ¯IRx)dx]=F1 exp(−L1μt1−L1μ¯IR)(exp(L1μt1+L1μ¯IR)−1)(μt1+μ¯IR)+C2 exp(−L1μ¯IR)(exp(L1σ1)−exp(L1μ¯IR))(σ1−μ¯IR)+C3 exp(−L1σ1−L1μ¯IR)(exp(L1σ1+L1μ¯IR)−1)(σ1+μ¯IR)+C4 exp(−L1σ2−L1μ¯IR)(σ2+μ¯IR)+F2 exp(−L1μ¯IR)(μt2+μ¯IR), (5)

where

$$F_{1}=(\frac{I_{0}\mu_{a1}(1-R_{1})\exp(-A_{s})}{2k_{1}(\sigma_{1}^{2}-\mu_{a1}^{2})})\;\text{and}\;F_{2}=(\frac{I_{0}\mu_{a2}(1-R_{1})\exp(-A_{s})\exp(-\mu_{t1}L_{1})}{2k_{2}(\sigma_{2}^{2}-\mu_{a2}^{2})}).$$

Here, ${\overset{¯}{\mu }}_{\mathrm{IR}}$ is the spectrally weighted IR absorption (emission) coefficient within layer 1. Following a temporal impulse excitation, the resulting transient temperature distribution is obtained from the inverse Fourier transform of Eq. (5):

S(t)=12π∫−∞∞S^(ω)eiωtdω. (6)

Due to the need for eigenvalue computations, eigenfunction expansions, and the intricate aspects of inverting Laplace or Fourier transforms, the frequency domain approach, using Eq. (6) and subsequent inverse Fourier transformation calculations, offers a less computationally intensive solution. In this inversion, from a sequence of N data points, there are N/2 useful frequencies. The sampling rate is given as

$$f_{s}=N\Delta f=\frac{1}{\Delta t},$$ (7)

where Δt and Δf are the sampling intervals in time domain and frequency domain, respectively. The inverse fast Fourier transform algorithm available in MATLAB^®^ allows for more effective processing of the signal. To ensure the appropriate acquisition of data points N for a specified maximum frequency in the photothermal wave field spectrum, denoted as $f_{\max}$, it is necessary to adhere to a sampling rate $f_{s}$ that meets the Shannon–Nyquist criterion, $f_{s}\geq 2f_{\max}$.

## 3 Experimental Methodology

**Fig. 2:**
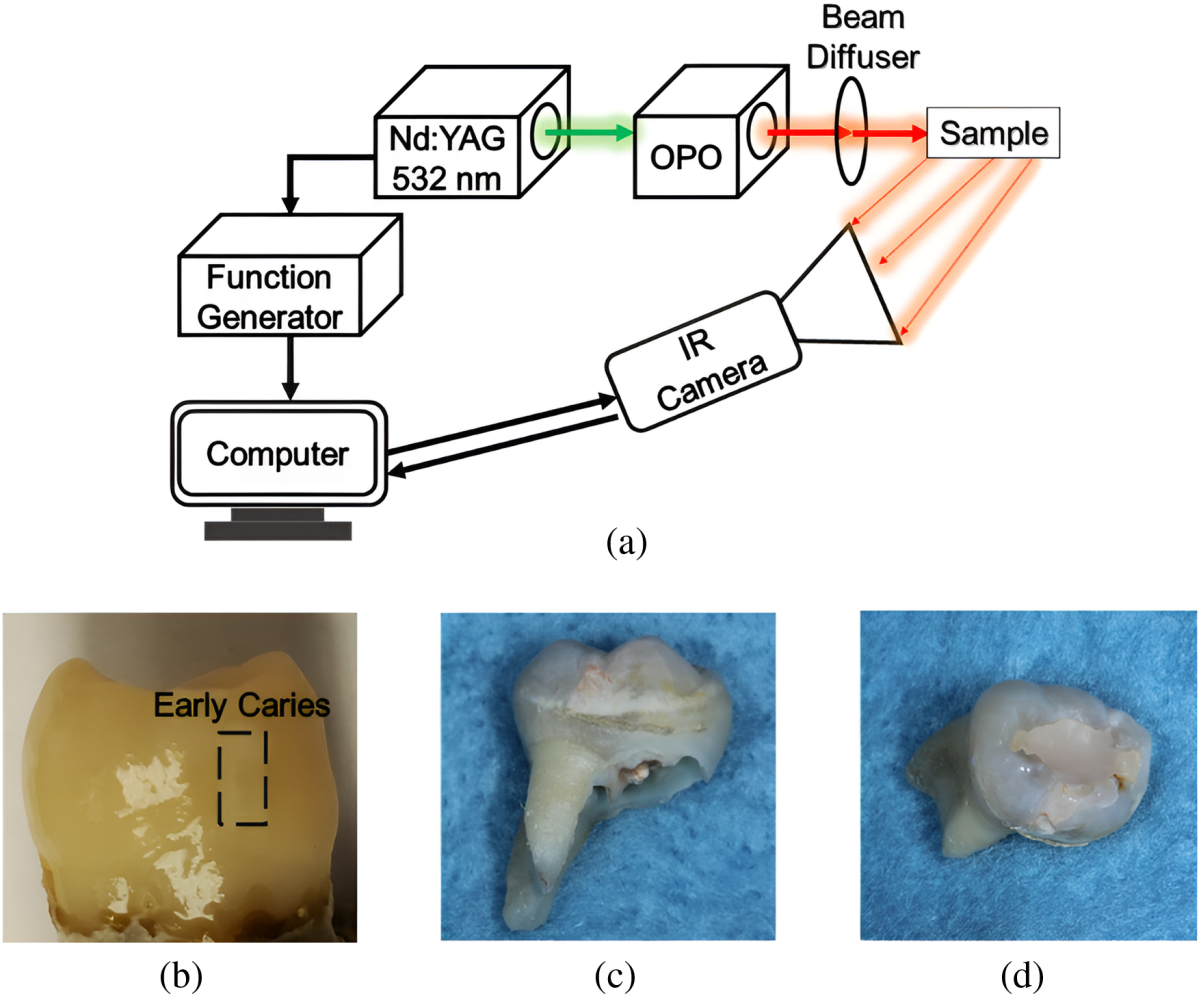
(a) Experimental configuration of multispectral photothermal system and the photograph of (b) sample 1 with artificially induced early caries, and (c) sample 2 front surface and (d) occlusal surface in which the tooth filling can be seen visually.

A Q-switched Nd:YAG laser (Surelite OPO Plus SLIII-10, Continuum, San Jose) generated 5-ns laser pulses at 10-Hz repetition rate which is the frequency of its flashlamp discharge [Fig. 2(a)]. The laser pulse was frequency doubled to 532 nm, which then pumped the OPO. The OPO output wavelength was tunable from 675 to 1000 nm and was controlled by a designated software for the crystal motor to achieve the desired wavelength. The experimental setup included an IR camera (A6700sc, FLIR, USA, 3 to 5  μm spectral response), which recorded the thermal evolution of the target sample following exposure to laser irradiation. The laser beam passed through a diffuser (ED1-C20, Thorlabs Inc., New Jersey) that homogenized and expanded the beam diameter to 20 mm. The camera image covered an area of 1.1  cm×0.88  cm. The frame rate of the camera was set to $f_{s}=104\;\mathrm{Hz}$, which recorded a video on its buffer in the FLIR format. Each frame of the video was exported as CSV file containing the photothermal transient data for each image pixel. Two extracted teeth samples were chosen in this study. Sample 1, a healthy extracted tooth, was used as a control reference sample to investigate early caries formation and progression in enamel; the visual photograph of the sample is shown in Fig. 2(b). To closely mimic the natural formation of dental caries, a treatment protocol using highly cariogenic bacteria was used to produce a carious lesion. The bacterial-induced artificial caries were limited to a rectangular section on the smooth surface of the tooth for a total exposure time of 8 days to induce early caries. Sample 2 was selected as a representation of a real-life sample with interior filling and several naturally occurring caries lesions on the tooth surface. Images depicting the tooth, including its occlusal surface highlighting the filling, as shown in Figs. 2(c) and 2(d).

Four OPO wavelengths were used to illuminate these samples: 675, 700, 750, and 808 nm. For every desired wavelength, a single pulse illuminated the sample using the single-shot operation mode and the IR camera recorded the thermal infrared response of the medium at the front surface. Because the energy per pulse of OPO wavelengths decreases with increasing wavelength, especially at 808 nm, we also conducted experiments with an 808 nm diode laser (Jenoptic JOLD- 120-QPXF-2P), which has a better power stability and is controlled by a laser driver (PCO-6131, Directed Energy, Colorado) to allow the user to select the desired laser pulse width (>10  ms) and repetition frequency. The laser output was passed through a collimator (F22SMA-B, Thorlabs Inc., New Jersey), and the same diffuser was used to expand the beam.

## 4 Results

### 4.1 Model Simulations

Transient temperature distributions of healthy and demineralized tooth geometries were simulated in MATLAB software using Eq. (6). To clearly distinguish parameters in healthy and demineralized tooth geometries, the subscript “j” is used alongside each parameter, such as $\alpha_{1,j}$. The variable j, with values 1 or 2, is used in simulation plots and in their discussion to represent healthy teeth geometry (j=1) and demineralized teeth geometry (j=2), respectively. For instance, $\alpha_{1,1}$ refers to the thermal diffusivity of layer one in healthy teeth geometry, which is the healthy enamel. On the other hand, $\alpha_{1,2}$ pertains to the thermal diffusivity of layer one in demineralized teeth geometry which comprises of demineralized enamel.

**Fig. 3:**
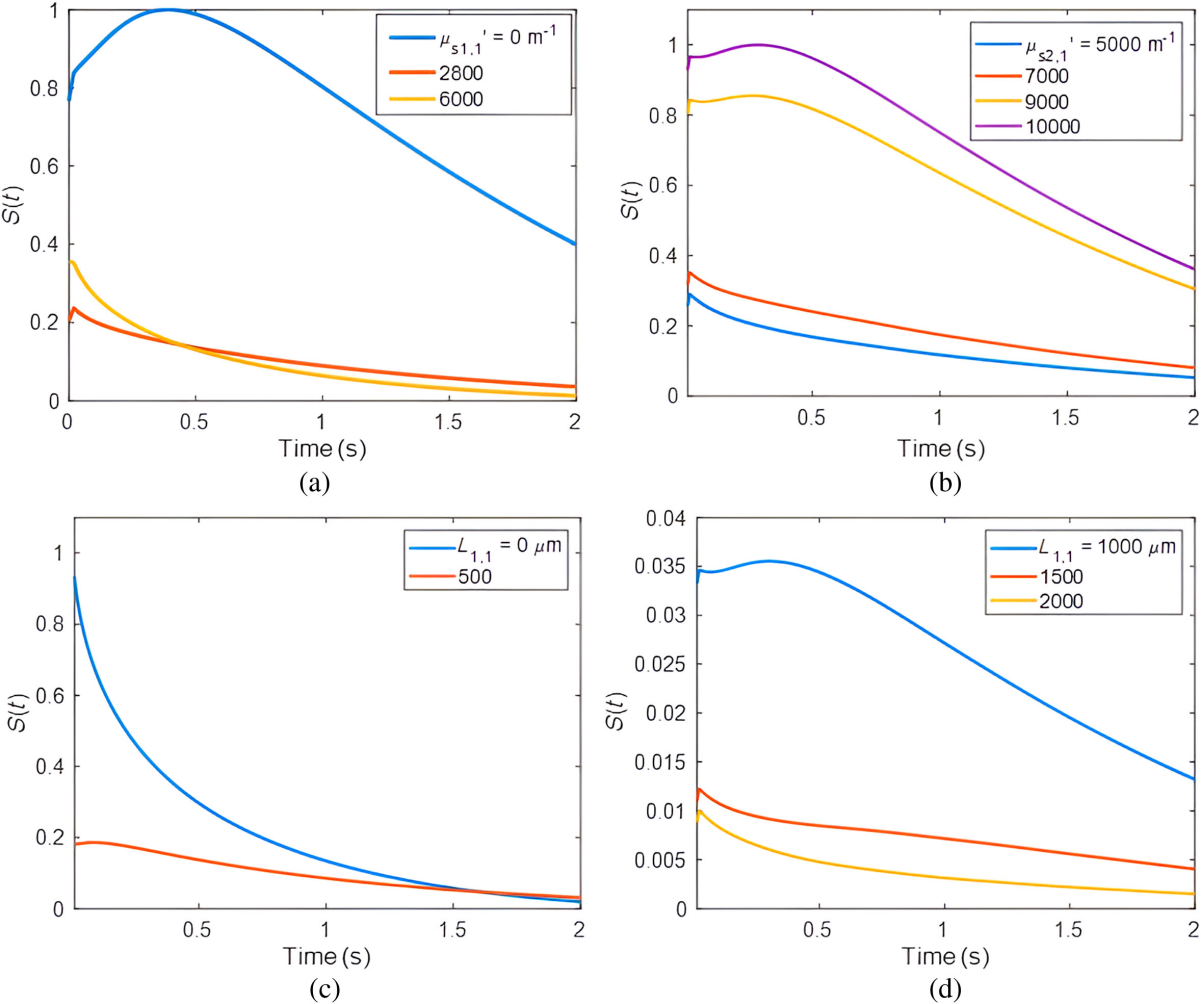
The effects of various parameters on theoretical signal profiles of Eq. (6) for healthy enamel: varying (a) the reduced scattering coefficient of the thin healthy enamel with other parameters fixed; and (b) the reduced scattering coefficient of semi-infinite dentin layer with other parameters fixed; and varying the thickness of enamel, $L_{1,1}$ (c) 0, 500  μm and (d) 1000 to 2000  μm.

**Fig. 4:**
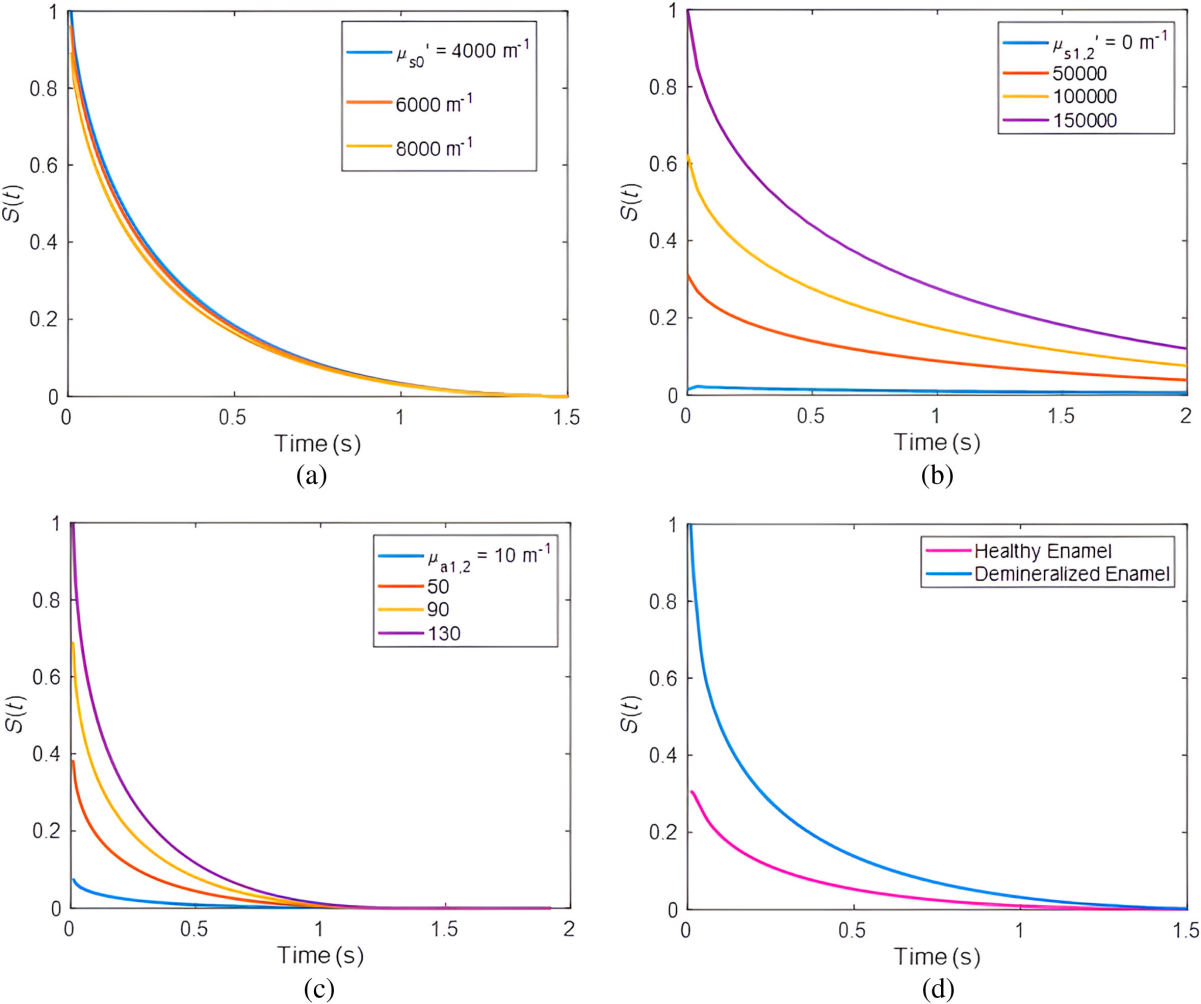
The effects of various parameters on theoretical signal profiles of Eq. (6) for a demineralized tooth: varying (a) the reduced scattering coefficient of the thin intact layer with other parameters fixed; (b) the reduced scattering coefficient of the demineralized enamel layer; (c) the absorption coefficient of the finite demineralized enamel layer with other parameters fixed; and (d) comparison between signals of healthy and demineralized tooth.

The thermal and optical parameters utilized in these simulations were acquired from the data extracted from other sources which also investigated the optical and thermal attributes of teeth.^17^^–^^20^ To simulate healthy tooth, the first layer comprised enamel, while the underlayer was composed of dentin. For enamel and dentin, the thermal conductivity and diffusivity remained constant at $\alpha_{1,1}=4.2\times 10^{-7}\;m^{2}{\cdot s}^{-1}$ $k_{1,1}=0.9\;W\cdot m^{-1}\cdot K^{-1}$, and $\alpha_{2,1}=1.8\times 10^{-7}\;m^{2}\cdot s^{-1}$ $k_{2,1}=0.6\;W\cdot m^{-1}\cdot K^{-1}$, respectively. The water infrared absorption (${\overset{¯}{\mu }}_{\mathrm{IR}}=120,000\;m^{-1}$) was used as the average value of the infrared absorption in both geometries. The thermal diffusivity and conductivity used for air interface were $\alpha_{g}=1.84\times 10^{-5}\;m^{2}\cdot s^{-1}$ and $k_{g}=2.62\times 10^{-2}\;W\cdot m^{-1}\cdot K^{-1}$, respectively. For healthy tooth, Fig. 3(a) shows the thermal transient signals with varying $\mu_{s1,1}^{\prime }$ while $\mu_{a1,1}=50\;m^{-1}$, $\mu_{a2,1}=300\;m^{-1}$, $\mu_{s2,1}^{\prime }=2000\;m^{-1}$, $L_{1,1}=1\;\mathrm{mm}$. The simulation results show that in the presence of zero or small reduced scattering coefficient in healthy enamel layer, the signals exhibit a dominating delayed peak due to the strong heat flow from the dentin layer and due to its higher reduced scattering coefficient and absorption properties compared to healthy enamel. By keeping the optical and thermal properties of the enamel layer constant with $\mu_{s1,1}^{\prime }=2800\;m^{-1}$, $\mu_{a1,1}=50\;m^{-1}$, $L_{1,1}=1\;\mathrm{mm}$ and only increasing $\mu_{s2,1}^{\prime }$ in Fig. 3(b), it is evident that the thermal transient signal, coupled with the rise in the delayed peak intensity, start to increase. This suggests a corresponding contribution of conduction and radiation heat transfer from the dentin layer, aligning with its increased scattering properties. Figures 3(c) and 3(d) show the thermal transient signal from the enamel-dentin medium when the thickness of enamel, $L_{1,1}$, increases between 0 and 2000  μm and all other parameters remain constant. The heat produced in layer 2 by dentin absorption is dominant at small values of $L_{1,1}$. Moreover, when the enamel thickness is increased, the conductive heat flux reaching the IR detector decreases, as the photothermal response from the recessed dentin layer diminishes. To investigate the theoretical signal profiles of the demineralized tooth area, the thermal conductivity and diffusivity of the demineralized enamel and the healthy enamel underneath remained constant at $\alpha_{1,2}=4.2\times 10^{-7}m^{2}\cdot s^{-1}$ $k_{1,2}=0.9\;W\cdot m^{-1}\cdot K^{-1}$, and $\alpha_{2,2}=4.2\times 10^{-7}\;m^{2}\cdot s^{-1}$, $k_{2,2}=0.9\;W\cdot m^{-1}\cdot K^{-1}$, respectively. Figure 4(a) shows the temperature transient result as the reduced scattering coefficients of the thin attenuating overlayer, $\mu_{s0}^{\prime }$, increase for the demineralized tooth. All other optical parameters of the tooth remained constant: $\mu_{a0}=50\;m^{-1}$, $\mu_{s1,2}^{\prime }=157,000\;m^{-1}$, $\mu_{a1,2}=100\;m^{-1}$, $\mu_{s2,2}^{\prime }=6000\;m^{-1}$, $\mu_{a2,2}=50\;m^{-1}$, $L_{1,2}=3\times 10^{-4}\;m$. Here, the PPTR signal decreased in the presence of higher reduced scattering of the thin overlayer because more of the laser light scattered and less absorbed by other layers within the sample. Examples of transient temperature distribution results for various reduced scattering coefficients of the demineralized enamel in layer 1, $\mu_{s1,2}^{\prime }$, are shown in Fig. 4(b). In the presence of increasing scattering in the demineralized layer, the signal amplitude increased and the photon fluence became more localized closer to the surface which reduced the optical flux to the healthy enamel underlayer.

Higher scatter of the optical field in the demineralized layer of enamel results in shorter photon path lengths within the enamel, which is photothermally equal to a higher photon absorption probability closer to the surface and increases the generated PPTR signal.^15^^,^^21^ The effect of the absorption coefficient, $\mu_{a1,2}$, of demineralized enamel is shown in Fig. 4(c): increasing the absorption coefficient increased optical-to-thermal energy conversion in the underlayer which, in turn, increased the photothermal signal at the detector/camera. Theoretical signal profiles of transient temperature distributions of healthy versus demineralized enamel are shown in Fig. 4(d). For healthy enamel, the optical parameters were: $\mu_{a1,1}=50\;m^{-1}$, $\mu_{s1,1}^{\prime }=2800\;m^{-1}$, $\mu_{a2,1}=300\;m^{-1}$, $\mu_{s2,1}^{\prime }=28,000\;m^{-1}$, $L_{1,1}=1\;\mathrm{mm}$. In addition, for demineralized enamel, the optical parameters were $\mu_{a0}=50\;m^{-1}$, $\mu_{s0}^{\prime }=2800$, $L_{0}=1.8\times 10^{-5}\;m$, $\mu_{a1,2}=100\;m^{1}$, $\mu_{s1,2}^{\prime }=157,000\;m^{-1}$, $\mu_{a2,2}=50\;m^{-1}$, $\mu_{s2,2}^{\prime }=2800\;m^{-1}$, $L_{1,2}=3\times 10^{-4}\;m$. Here, the healthy tooth enamel has a smaller amplitude compared to the demineralized tooth. The demineralized enamel results suggest that there is a significant reduction in light intensity transmission and an increase in light scattering, especially at interfaces where light is reflected back. When light enters the tooth, it is scattered preferentially in the carious areas where the pore volume is large. The greater the light scattering in an area, the higher the probability of absorption in that area. As a result, the thermal waves that are generated in the porous regions will have greater amplitude than those generated in intact enamel.

### 4.2 Experimental Results

**Fig. 5:**
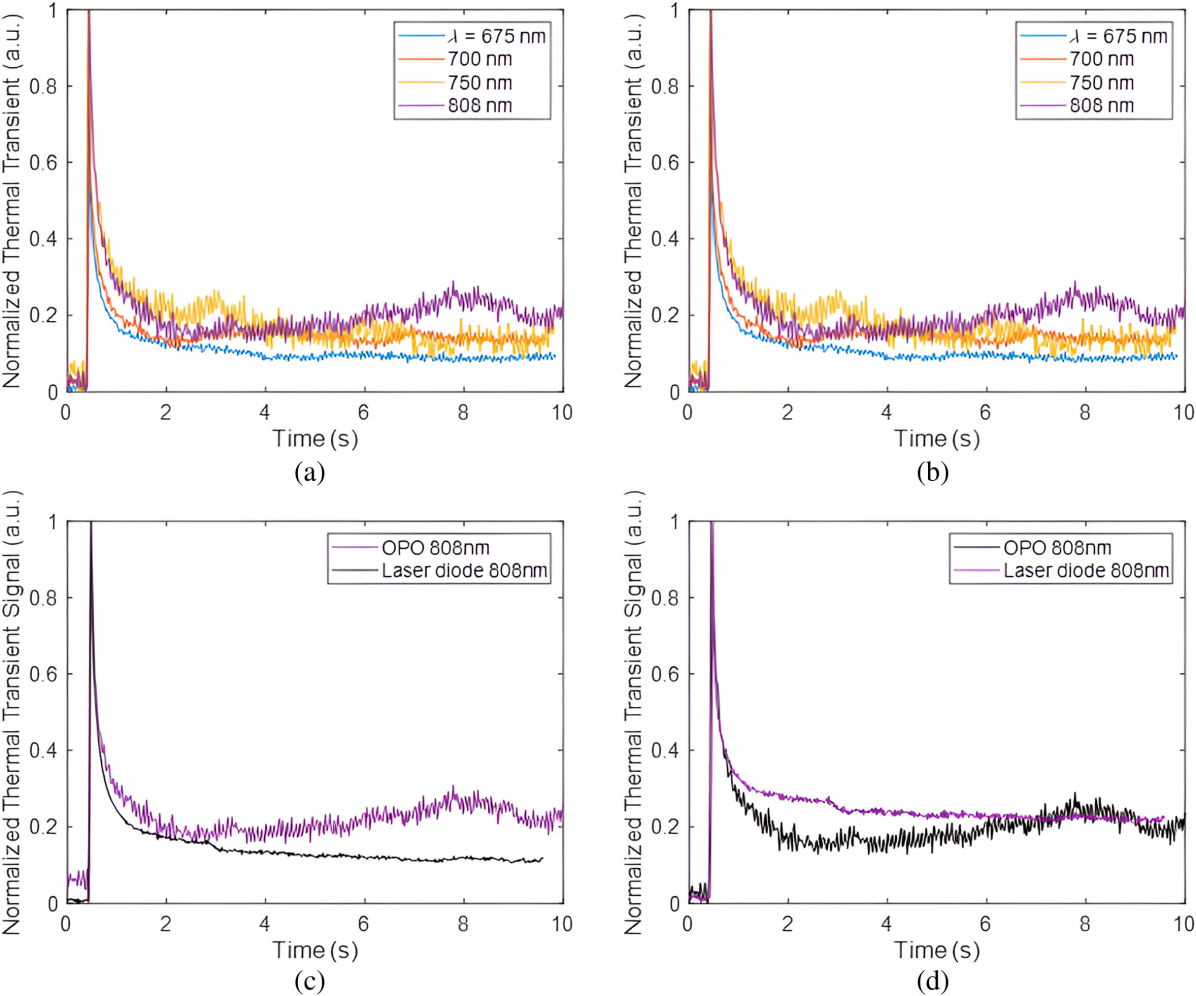
Normalized sample 1 experimental thermal transient signals for (a) healthy and (b) demineralized tooth pixel at pulsed Nd:YAG laser OPO wavelengths 675, 700, 750, and 808 nm. The comparison between the thermal transient signals of 808 nm OPO and 808 nm laser diode for (c) demineralized and (d) healthy enamel.

Figures 5(a) and 5(b) show the photothermal transient signals at every OPO wavelength for the healthy and demineralized areas of sample 1. The thermal transient signals shown in the figure were the responses of one pixel from the healthy and demineralized areas. A normalization technique was applied, involving the division of the entire signal by its initial (peak) magnitude. This eliminated the need to determine the intensity of the incoming laser beam and the calibration coefficients. It can be seen that in the normalized thermal transient plots of the demineralized area as a function of OPO wavelength, the 675 nm wavelength decayed fastest and the 808 nm decayed slowest.

This is due to the wavelength dependence of the scattering and absorption coefficients of the demineralized area such that at shorter wavelengths (or higher optical frequency) the demineralized area scatters and absorbs more strongly than at longer wavelengths. Here, caries is at its earliest stages, hence it is a near-surface feature. Shorter wavelengths tend to capture such shallow features due to the shorter thermal diffusion lengths involved due to the highly scattering nature of the lesions which results in near-surface photon localization. Longer OPO wavelengths can therefore penetrate deeper into the substructure and display a slower decaying thermal transient upon exposure to laser light. In the case of the normalized thermal transient signals for healthy enamel, the signals from each OPO wavelength seemed to be closely similar.

The signals from the 808 nm OPO excitation displayed a similar decaying pattern but with a deeper penetration capability of this wavelength compared to other wavelengths emitted by the OPO. Nonetheless, at 808 nm, a reduction in OPO energy led to fluctuating thermal emission signal which was assumed to be coupled with temperature fluctuations in the environment in which the experiment was performed. To confirm that this indeed constituted background noise and was not linked to the properties of the sample layered and optothermal characteristics, an experiment was performed using an 808 nm laser diode which exhibited significantly superior power stability. Figures 5(c) and 5(d) show the plots of the thermal transients of the two lasers at 808 nm. It is evident that the 808-nm laser diode exhibited a smooth decaying behavior without showing any major fluctuations in the thermal transient signal. However, the signal decay rate due to this laser diode differed from that of the 808 nm OPO. This variance can be attributed to the fact that the laser diode’s minimum pulse width was configured at 10 ms, indicating that it released energy over a longer duration compared to the pulsed laser with a pulse width of 5 ns.

**Fig. 6:**
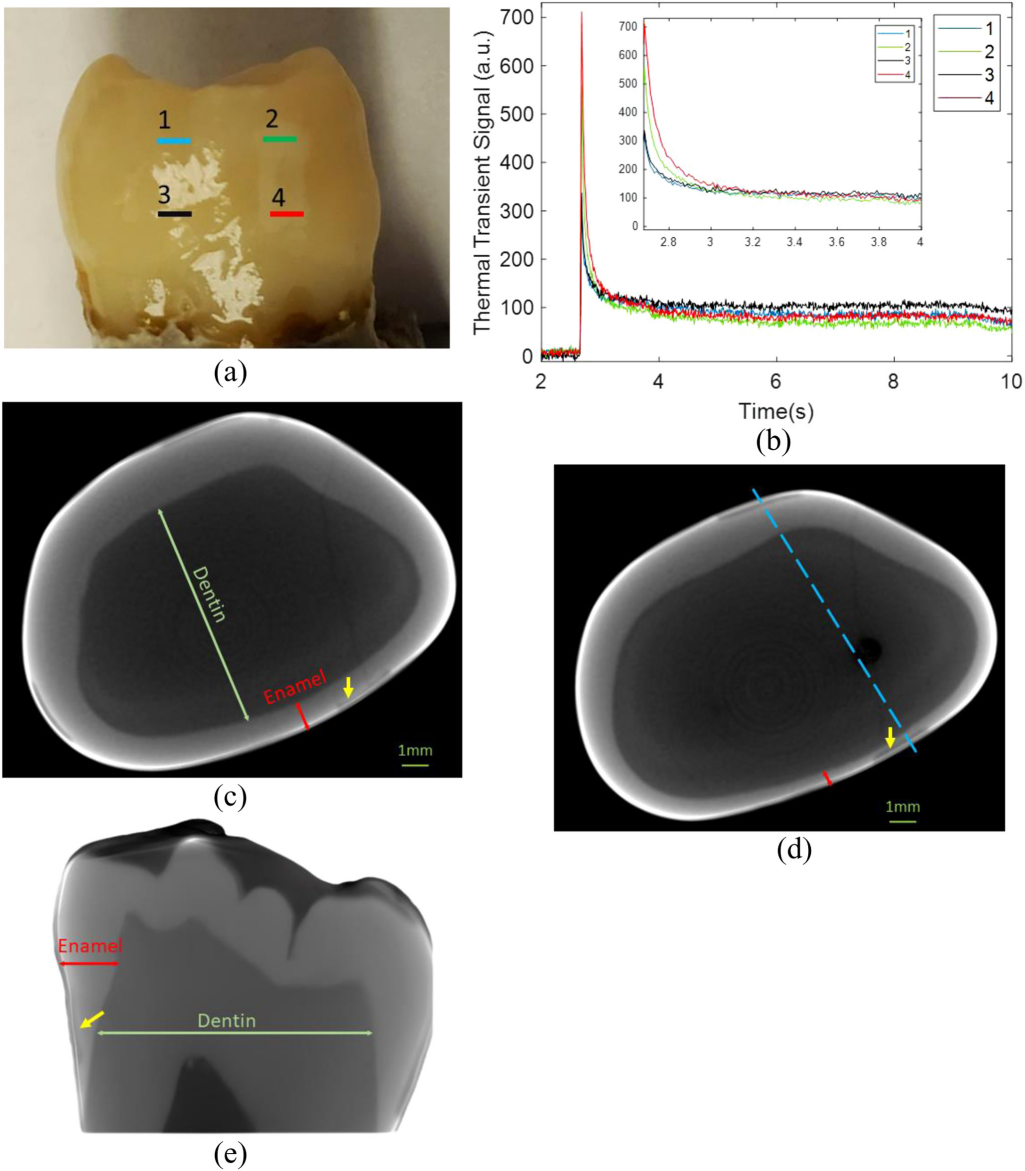
(a) Visual photograph of the tooth marked by four different locations on one surface of the sample. Locations 1 and 2 belong to healthy and demineralized enamel on the same axial plane near the cusp of the tooth, and locations 3 and 4 mark the healthy and demineralized enamel on the same axial plane close to the cementoenamel junction where enamel is thinnest; (b) corresponding thermal transient signal plots are shown for each location at 675 nm OPO wavelength. The μCT cross-sectional slices of the tooth depict locations (c) 1 and 2, and (d) 3 and 4. The thicknesses of enamel and dentin change at different locations of the tooth. This is shown by the (e) μCT sagittal view at the cross-section indicated by the dashed blue line in (d). The yellow arrow shows the demineralized artificial caries.

**Fig. 7:**
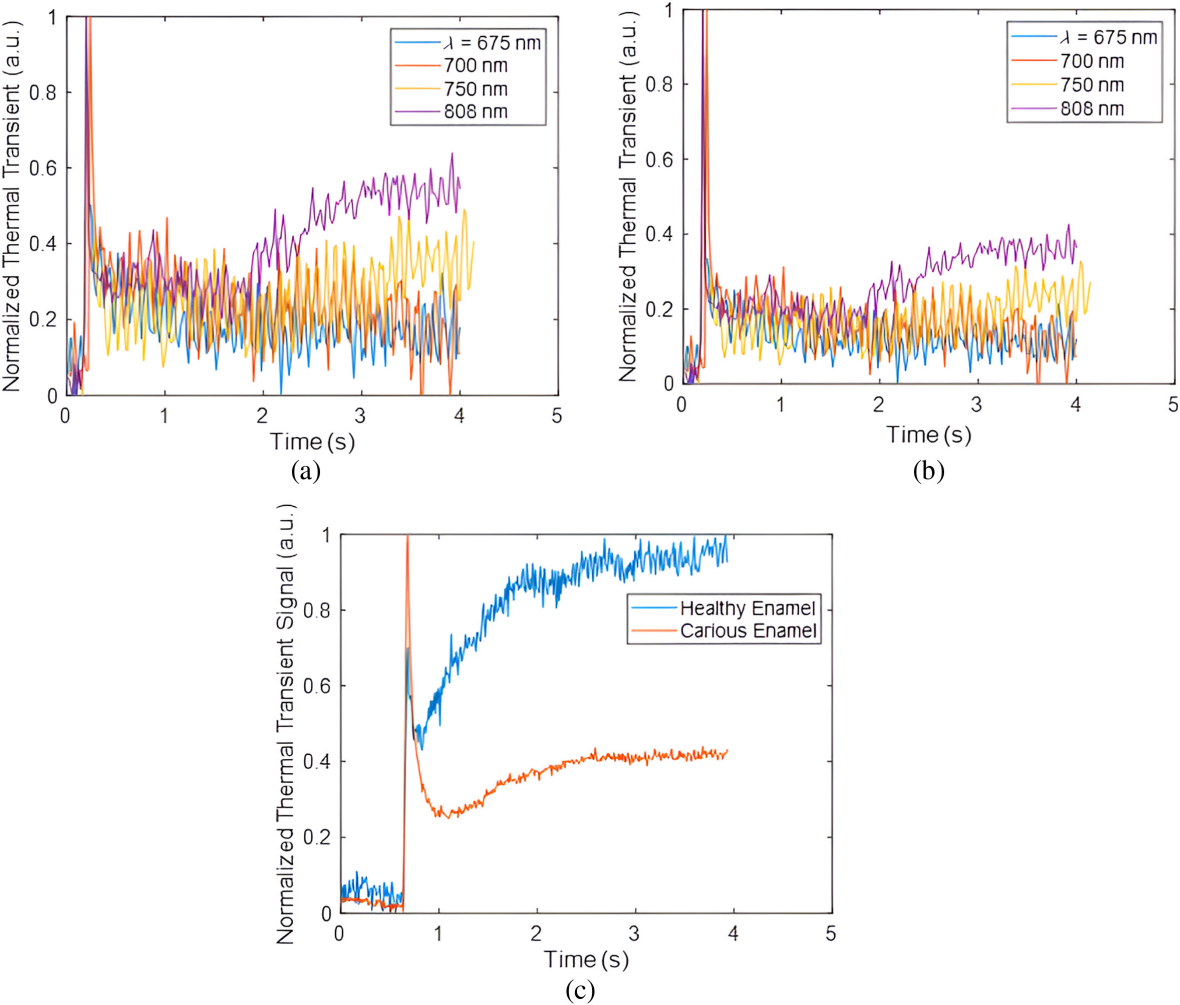
Sample 2 experimental thermal transient signals for (a) healthy and (b) carious area at pulsed Nd:YAG laser OPO wavelengths 675, 700, 750, and 808 nm; (c) the thermal transient plots of the 808 nm laser diode excitation are also shown for comparison purposes.

**Fig. 8:**
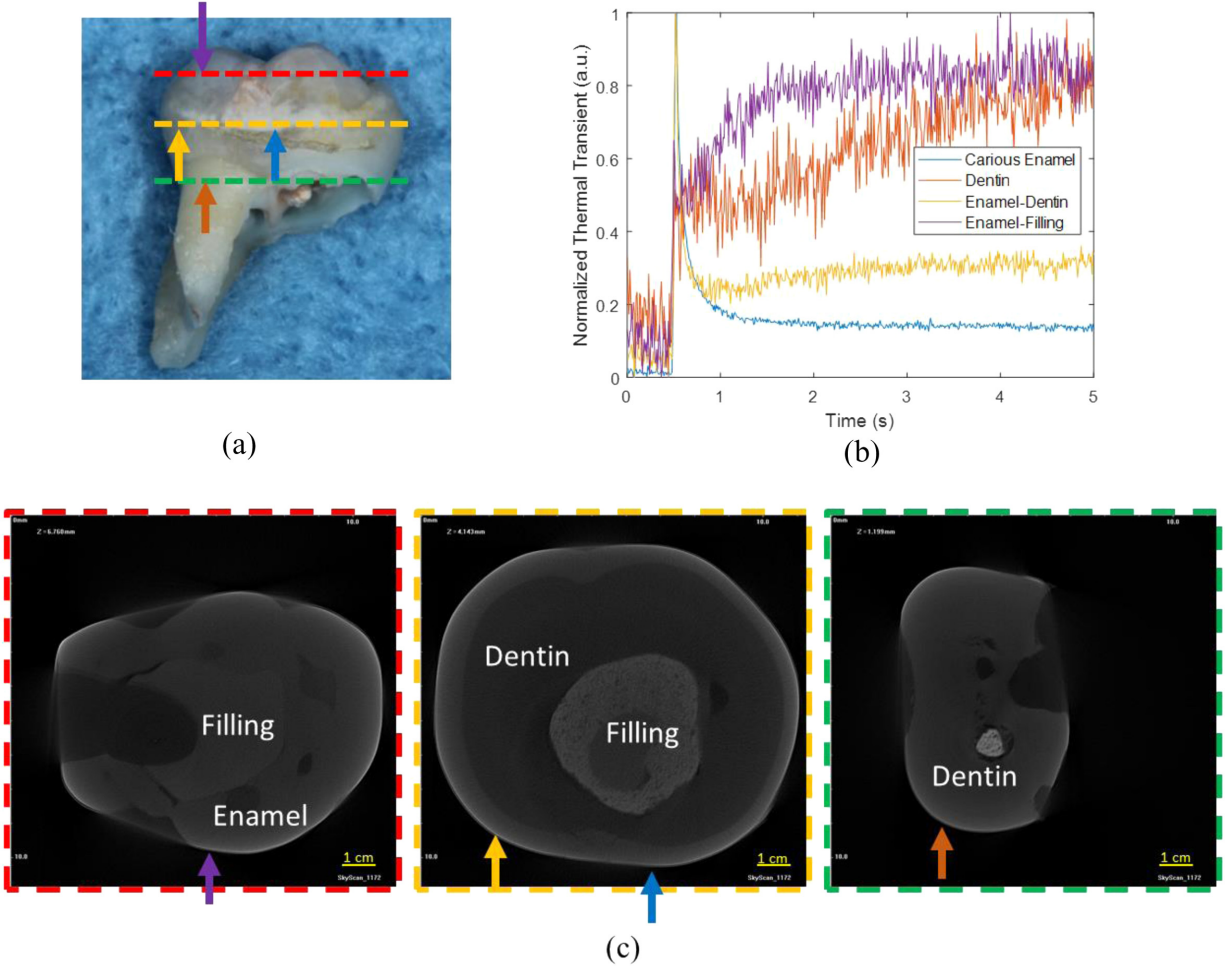
(a) A photograph of sample 2, identified by three distinct cross-sectional lines on the surface on the tooth, along with four arrows situated at various points where photothermal transients were obtained. (b) Plots depicting thermal transient signals are displayed for every point indicated by the color-coded arrows, captured using the 808 nm laser diode. (c) The μCT slices of the tooth at the cross-sections indicated by dashed colored lines in (a) along with the locations of the marked arrows on the sample.

For signals originating in sound enamel, the degradation of the 808 nm laser diode output was more gradual due to its ability to capture a greater amount of conductive and radiative heat transfer from the dentin layer. In addition to the normalized data as a function of wavelength, the normal thermal transient plots are shown in Figs. 6(a) and 6(b) at four different regions of the tooth at 675 nm wavelength. While wavelength affects the shape of the thermal transient signal, it is important to note that at different locations on the tooth, the thermal transient plot can be different. As an example, locations 1 and 2 refer to the sound enamel and demineralized enamel, respectively, in proximity to the tooth’s cusp. Meanwhile, positions 3 and 4 depict healthy and demineralized enamel, respectively, near the cementum where enamel is thinnest. The demineralized enamel adjacent to the cusp (location 2) exhibited smaller amplitude in comparison to the demineralized enamel at location 4 near the cementum. However, it also exhibited faster decay. This implies that the demineralized area close to the cementum, at location 4, might have also been influenced by the more absorbing and scattering dentin region. The healthy enamel regions adjacent to the cusp and cementum (i.e., locations 1 and 3), both exhibited similar amplitudes in their thermal transient signals. However, at location 3, which is closer to the cementum, the decay occurred at a slower rate compared to location 1. This can be attributed to contributions from the underlying dentin layer. Micro-computed tomography (μCT) was conducted to confirm the presence and extent of the demineralized region [as can be seen by yellow arrows in Figs. 6(c)–6(e)] and other features and characteristics of the tooth. Figure 6(c) shows the μCT image slice that corresponds to the cross-sectional view of the tooth encompassing locations 1 and 2. Likewise, Fig. 6(d) corresponds to the μCT cross-sectional slice containing locations 3 and 4. One prominent characteristic that can be seen in these μCT images is the change in enamel thickness which can affect the thermal transients. In Fig. 6(e), a three-dimensional cross-sectional μCT image of a sample is displayed, obtained by cutting along the indicated blue lines in Fig. 6(d). The change in enamel thickness measures around 600  μm is readily apparent from cusp to cementum for this specific sample surface. The thermal transient signals from sample 2 at various OPO wavelengths for healthy and carious pixels are shown in Figs. 7(a) and 7(b). For this sample, shorter wavelength signals exhibited a gradual decrease, while longer wavelength signals showed a delayed rise in signal amplitude. To further validate this observation, the 808 nm laser diode was used to irradiate the sample, and the thermal transient signals from the same healthy and carious pixels were plotted [Fig. 7(c)]. We opted to utilize the 808-nm laser diode for the quantitative analysis of this particular sample due to its improved signal-to-noise ratio. Subsequently, thermal transient signals were plotted at distinct positions on the tooth. In Fig. 8(a), the sample’s visual image features four variably colored arrows, each associated with its respective thermal transient signal as shown in Fig. 8(b). These varying transient signals served as valuable indicators for subsequently extracting thermal and optical characteristics of the tooth. They show both the magnitude of initial rise and the subsequent decay and beyond in each signal. As an example, in the thermal transient plot of Fig. 8(b) showing the dentin response, the initial magnitude was notably minimal and practically insignificant. This observation indicated a substantial reduction in contributions from the enamel layer. Conversely, in the case of the carious region, the strong thermal transient signal displayed a rapid monotonic decay pattern. This behavior signified that the influences stemming from the underlying characteristics were entirely concealed by the presence of the carious lesion. The μCT slices shown in Fig. 8(c) were acquired from the cross-sections indicated by colored dashed lines in Fig. 8(a). The μCT slices display greatly varying tooth layers and internal filling areas at different positions. This information became valuable for subsequent quantitative measurements and imaging of the sample optothermal characteristics.

## 5 Multiparameter Computation and Quantitative Imaging

To obtain the thermal and optical characteristics of these dental samples, a method involving multiparameter fitting was implemented utilizing a simplex downhill minimization algorithm.^17^^,^^22^^–^^24^ A comprehensive explanation of this fitting methodology, which was also utilized in assessing the optical and thermal properties of dental tissue in previous research, can be found elsewhere.^12^^,^^24^^,^^25^

In this multiparameter fitting of sample 1, it was assumed that the intact surface layer of the demineralized enamel has the same optothermal properties as the healthy semi-infinite enamel underlayer. In addition, the values fitted for the healthy enamel geometry were used as known parameters for both the healthy semi-infinite underlayer and the intact surface layer of the demineralized enamel. To ensure uniqueness in parameter fitting, an added approach that proved beneficial involved selecting thermal transient signals from regions adjacent to the tooth cusp and in proximity to the cementum. In the vicinity of the cusp, there exists only enamel, allowing the use of a single-layer theory to determine enamel properties. Likewise, in the proximity to the cementum, only dentin is present, enabling a similar approach for dentin characterization. The μCT images were also used for validation purposes and enamel thickness measurements. In real-world scenarios where enamel thickness is unknown or where μCT images are unavailable, it would be appropriate to consider enamel thickness as a fitting parameter. The first step consisted of using the data from healthy enamel close to the tooth cusp and dentin near the cementum, while assuming a single semi-infinite layer. The value ranges used, seen in Table 1, were then split into 5 to 10 uniform intervals. For each combination and parameter, the best fits were obtained. The parameters derived from this initial fitting, within the 5 to 10 division range, were averaged and used as input values for a second fit using the same number of divisions (5–10) between the limits. This averaging process was repeated until the set of optical and thermal parameters became independent of the number of divisions between the limits. Afterward, a thermal transient signal situated at a pixel location within sound enamel was selected, encompassing both the enamel and underlying dentin. The enamel thickness at this specific pixel was found by referencing the associated μCT image and best fitting was carried out utilizing a two-layer configuration.

**Table 1:** Fixed upper and lower limits of the fundamental parameters defined for the multiparameter fitting of sound and demineralized enamel.

Physical parameters layer	Sound enamel^a^		Demineralized enamel^b^	
	Lower limit	Upper limit	Lower limit	Upper limit
$\mu_{s0}^{\prime }$ ($m^{-1}$)	—	—	1000	6000
$\mu_{a0}$ ($m^{-1}$)	—	—	1	100
$L_{0}$ (μm)	—	—	5	200
$\mu_{s1}^{\prime }$ ($m^{-1}$)	1000^26^	6000^9^	1000^9^	157,000^27^
$\mu_{a1}$ ($m^{-1}$)	1	100^9^	1	150
$\mu_{s2}^{\prime }$ ($m^{-1}$)	8400^9^	28,000^9^	1000	6000
$\mu_{a2}$ ($m^{-1}$)	300^28^	500	1	100
$\alpha_{1}$ ($m^{2}/s$)	$4.2\times 10^{-7}$ ^20^	$4.7\times 10^{-7}$ ^29^	$2.0\times 10^{-7}$ ^20^	$7.7\times 10^{-7}$
$\kappa_{1}$ (W/m·K)	0.77^20^	0.93^18^	0.1	0.93^18^
$\alpha_{2}$ ($m^{2}/s$)	$1.8\times 10^{-7}$ ^29^	$2.6\times 10^{-7}$ ^30^	$4.2\times 10^{-7}$	$4.7\times 10^{-7}$
$\kappa_{2}$ (W/m·K)	0.57	0.62	0.77^20^	0.93^18^
$H_{1}$ and $H_{2}$ ($W/m^{2}\cdot K$)	1	500	1	500

a Sound enamel: layer one, sound enamel; layer two, semi-infinite sound dentin.

b Demineralized enamel: layer one, demineralized enamel; layer two, semi-infinite sound enamel.

**Fig. 9:**
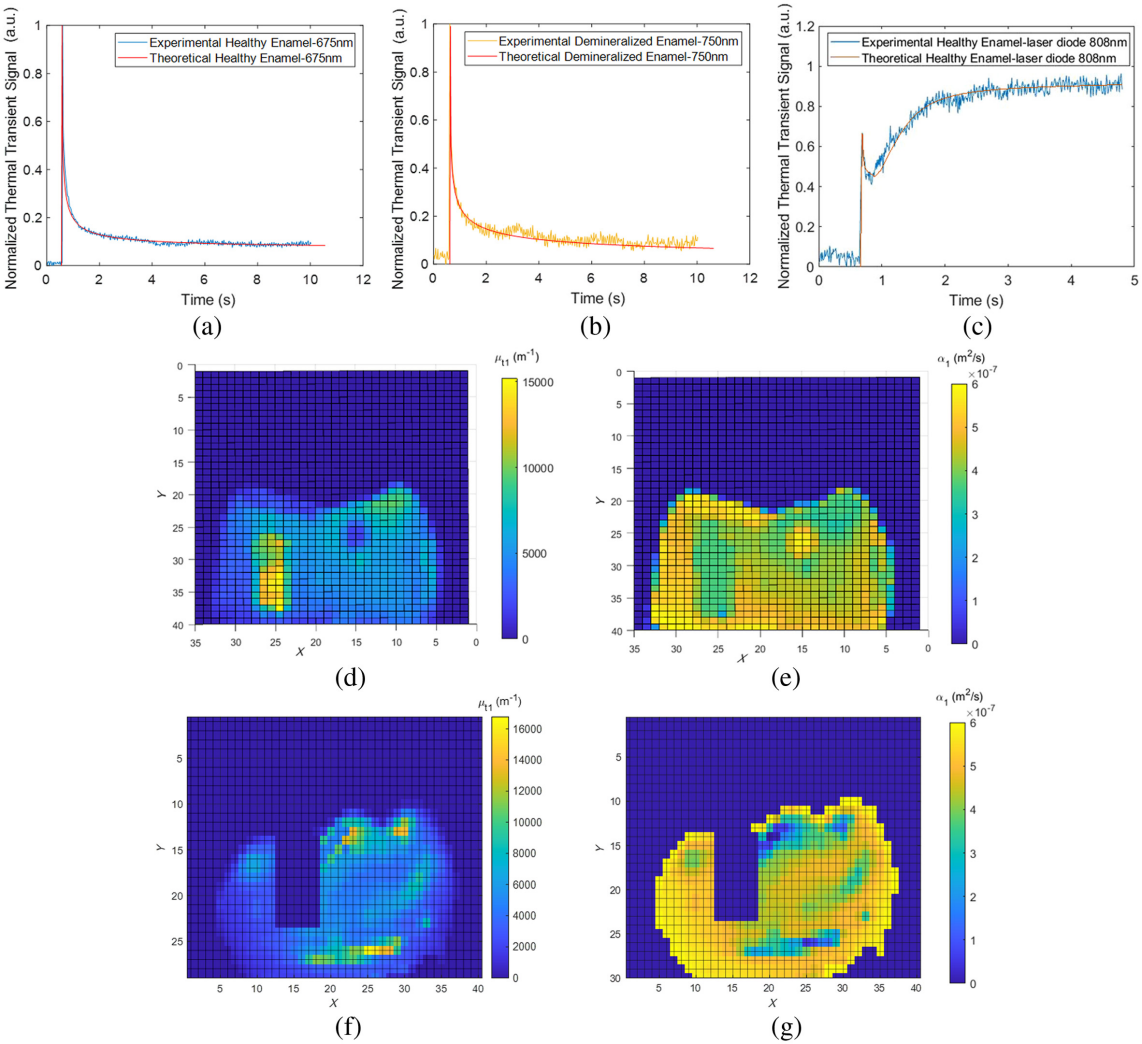
Examples of experimental and theoretical best fits for sample 1 healthy and demineralized enamel at (a) 675 nm and (b) 750 nm, respectively, and the healthy enamel fit with (c) 808 laser diode. Best fitted parameter values are shown in Tables 2 and 3. Derived pixel-by-pixel best-fitted parameter images of (d) optical transport coefficient image and (e) thermal diffusivity image of sample 1 for 700 nm OPO wavelength. (f) Optical transport coefficient image and (g) thermal diffusivity image of sample 2 for 808 nm laser diode. Note that, in the case of defective areas within the tooth, the demineralized geometry was employed. Consequently, the parameter representation on the scale bar displays general parameter names without specifying the associated geometry.

We deliberately chose pixels in which enamel thickness was easily quantifiable in the μCT images. The results from the adjustment exhibited slight fluctuations in the values for the enamel and dentin underlayers. This disparity can be attributed to the non-uniform nature of teeth as samples, causing the characteristics of enamel and dentin to shift based on the location on the tooth. Average fitted optical and thermal properties of healthy and demineralized enamel in a two-layer medium and their standard deviations are shown in Tables 2 and 3, respectively. The results showed excellent agreement between the experimental and theoretical data when fitting the multiple parameters of both healthy and demineralized enamel, as shown in Fig. 9. The fit of the 675, 700, and 750 nm was very good. As mentioned, at 808 nm, due to a decrease in the OPO energy, variations in room temperature led to fluctuations in the sample thermal emission, resulting in background noise characterized by fluctuating signals. Consequently, the diode laser was used for those best fits. Overall, the demineralized enamel compared to healthy enamel showed a significantly higher signal due to the fact that in the presence of a higher-reduced scattering coefficient medium in the demineralized region, resulting in higher photon confinement and optical-to-thermal energy conversion in the near-surface region, while lessening the influence of the underlying layers. In relation to the initial constraints defined for the parameters, it is important to emphasize that all parameter limits had the flexibility to adjust within the defined range encompassing the minimum and maximum literature values, as specified in Table 1. Nevertheless, limitations on enamel thickness were determined based on the established μCT measurements. Parameters, such as the optical absorption and reduced scattering coefficients, along with thermal diffusivities and conductivities, stood out as the most consistently influential values. On the other hand, measuring the heat transfer coefficients exhibited a notably higher degree of variability. This shows that in the process of fitting multiple parameters there is a variable degree of reliability and only the most reliable parameters should be selected while other less dependable ones given less weight or even ignored. To further evaluate the effectiveness of the multiparameter fitting approach, several pixels from the healthy enamel of a tooth were selected. Without relying on μCT measurements, best fits were obtained for various optical and thermal properties along with enamel thickness. The resulting enamel thickness, when compared to its actual thickness obtained through μCT scanning, produced a residual error of 3.32%. This is of utmost importance because in practical/clinical situations there are no data available relying on destructive techniques, such as μCT, and clinician dependence on non-invasive testing methods to extract the optical and thermal characteristics of teeth is expected to be total. The photothermal transient data from the experiments were captured using the IR camera, affording the possibility to gather optothermal characteristics across the entire surface of the tooth sample. Up until now, the process of fitting photothermal transients at distinct wavelengths was solely applied to individual pixels of the IR camera situated at different locations on the tooth surface. However, due to the high computational demands and lengthy calculation times associated with the fitting procedure, a 16×16-pixel averaging technique was employed to decrease the pixel count, subsequently deriving the properties for each averaged pixel. Furthermore, we opted to focus on a single optical OPO wavelength, specifically choosing 700 nm as the optimal wavelength for our purposes. Figures 9(d) and 9(e) constitute quantitative images showing plots of optical transport coefficient and thermal diffusivity of layer one of sample 1, respectively. It can be seen from these quantitative images that the thermal diffusivity of the demineralized area is smaller compared to other regions while the optical transport coefficient increases. This is as expected because low diffusivity results in local thermal confinement and higher temperatures.

**Table 2:** Average fitted optical and thermal properties of sample 1 healthy enamel in a two-layer medium and their standard deviations.

Healthy enamel										
	$\mu_{s1,1}^{\prime }$ ($m^{-1}$)	$\mu_{a1}$ ($m^{-1}$)	$\mu_{s2,1}^{\prime }$ ($m^{-1}$)	$\mu_{a2,1}$ ($m^{-1}$)	$\alpha_{1,1}$$m^{2}/s$)	$\kappa_{1,1}$ (W/m·K)	$\alpha_{2,1}$ ($m^{2}/s$)	$\kappa_{2,1}$ (W/m·K)	$H_{1,1}$ ($W/m^{2}\cdot K$)	$H_{2,1}$ ($W/m^{2}\cdot K$)
675 nm	7640 ± 20	46 ± 5	13,400 ± 100	360 ± 11	$4.5\pm 0.05\times 10^{-7}$	0.92 ± 0.08	$2.3\pm 0.03\times 10^{-7}$	0.64 ± 0.05	122 ± 68	80 ± 30
700 nm	7280 ± 20	43 ± 6	11,010 ± 150	360 ± 19	$4.5\pm 0.05\times 10^{-7}$	0.92 ± 0.08	$2.3\pm 0.03\times 10^{-7}$	0.64 ± 0.05	231 ± 102	76 ± 68
750 nm	5410 ± 50	44 ± 6	13,200 ± 300	330 ± 10	$4.5\pm 0.05\times 10^{-7}$	0.92 ± 0.08	$2.3\pm 0.03\times 10^{-7}$	0.64 ± 0.05	131 ± 95	101 ± 35
800 nm	5200 ± 90	37 ± 9	10,400 ± 600	300 ± 30	$4.5\pm 0.05\times 10^{-7}$	0.92 ± 0.08	$2.3\pm 0.03\times 10^{-7}$	0.64 ± 0.05	301 ± 141	140 ± 71
808 nm laser diode	4700 ± 10	45 ± 2	8600 ± 10	310 ± 30	$4.5\pm 0.05\times 10^{-7}$	0.92 ± 0.08	$2.3\pm 0.03\times 10^{-7}$	0.64 ± 0.05	101 ± 42	78 ± 30

**Table 3:** Average fitted optical and thermal properties of sample 1’s demineralized enamel in a two-layer medium and their standard deviations.

Demineralized enamel							
	$L_{0}$ (μm)	$\mu_{s1,2}^{\prime }$ ($m^{-1}$)	$\mu_{a1,2}$ ($m^{-1}$)	$\alpha_{1,2}$ ($m^{2}/s$)	$\kappa_{1,2}$ (W/m·K)	$H_{1,2}$ ($W/m^{2}\cdot K$)	$H_{2,2}$ ($W/m^{2}\cdot K$)
675 nm	68 ± 5	10,700 ± 100	45 ± 3	$3.7\times 10^{-7}\pm 0.04\times 10^{-7}$	0.56 ± 0.03	221 ± 53	60 ± 17
700 nm	68 ± 5	9800 ± 70	43 ± 5	$3.7\times 10^{-7}\pm 0.04\times 10^{-7}$	0.56 ± 0.03	123 ± 30	41 ± 12
750 nm	68 ± 5	6000 ± 40	46 ± 10	$3.7\times 10^{-7}\pm 0.04\times 10^{-7}$	0.56 ± 0.03	131 ± 45	53 ± 31
800 nm	68 ± 5	5100 ± 50	40 ± 10	$3.7\times 10^{-7}\pm 0.04\times 10^{-7}$	0.56 ± 0.03	140 ± 35	46 ± 16
808 nm laser diode	68 ± 5	4200 ± 15	43 ± 3	$3.7\times 10^{-7}\pm 0.04\times 10^{-7}$	0.56 ± 0.03	96 ± 21	58 ± 10

The multiparameter fits for sample 2 diverge from those for sample 1 because of the various distinct characteristics found on the tooth surface. These features include unevenly distributed caries across the tooth’s surface and a visibly damaged area on the tooth’s surface. According to the multiparameter fitting approach applied to sample 1, a handful of pixels were selected and the corresponding μCT cross-sectional image slices for these pixels were matched. This was done to validate the thicknesses and true existence of various features. When investigating the characteristics of enamel, a specific pixel close to the cusp was chosen situated toward the edge of the sample to avoid any interference from the filling, as shown in the μCT image of Fig. 8(a). Subsequently, the one-layer theory was used to derive the properties of the sound enamel in that pixel. A similar procedure was carried out in the case of dentin, as shown in Fig. 8(c). Another pixel was selected to depict the white caries and in the μCT image of Fig. 8(b); however, due to the fact that the depth of the white cavities was not precisely known, $L_{1}$ was used as an adjustable fitting parameter in this case. Regarding the presence of a thin intact layer at x=0, this carious region appeared by visual inspection to be advanced caries. Thus, the intact layer was assumed not exist for this sample. The resulting parameters obtained from these pixels are shown in Table 4 and an example of the optimal fit to the sound enamel of sample 2 is shown in Fig. 9(c) and the quantitative images of optical transport coefficient and thermal diffusivity of layer one is shown in Figs. 9(f) and 9(g), respectively. An area of this sample was excluded from the fitting process because it exhibited significant surface defects that were visible to the eye, and obtaining accurate fitting parameters proved to be challenging.

**Table 4:** Average fitted optical and thermal properties of sample 2’s various pixels on the tooth and their standard deviations.

808 nm laser diode							
	$L_{1}$ (μm)	$\mu_{s1}^{\prime }$ ($m^{-1}$)	$\mu_{a1}$ ($m^{-1}$)	$\alpha_{1}$ ($m^{2}/s$)	$\kappa_{1}$ (W/m·K)	$H_{1}$ ($W/m^{2}\cdot K$)	$H_{2}$ ($W/m^{2}\cdot K$)
Healthy enamel	—	2300 ± 10	21 ± 2	$4.6\pm 0.07\times 10^{-7}$	0.81 ± 0.05	23 ± 12	—
Healthy dentin	—	9200 ± 40	38 ± 4	$1.89\pm 0.07\times 10^{-7}$	0.61 ± 0.03	37 ± 8	—
Carious enamel^a^	102 ± 8	16,200 ± 34	101 ± 4	$2.65\pm 0.04\times 10^{-7}$	0.56 ± 0.06	45 ± 21	12 ± 4

a Demineralized enamel geometry was used to obtain the optical and thermal properties.

## 6 Conclusions

The developed theoretical framework explains the behavior of optical and thermal-wave depth profiles within multilayer dental structures. It measures the impact of optical and thermal characteristics via an inverse Fourier technique, as well as the interactions between these layers, on the patterns observed in depth profiles. Employing an IR camera to capture PPTR signals from two dental specimens selected for a number of subsurface features enabled us to obtain the optothermal characteristics of these samples based on pixel variations. PPTR experiments performed with an OPO and a laser diode along with validations using μCT, validated the theory in relation to multilayers. The incorporation of the fitting method in this study enhanced the reliability of the computational algorithm, yielding a dependable solution for accurately fitting multiple parameters related to both intact enamel and enamel caries lesions in multilayered structures. The degree of reliability of each of the fitted parameters was identified and led to retention of the thermal diffusivity, thermal conductivity, enamel thickness, and optical transport coefficient of the enamel layer as the key reliable parameters in quantitative pixel-by-pixel images. Finally, performing quantitative dental property measurements and imaging plays a crucial role in clinical practice because one can develop a calibration chart which, in collaboration with practicing dentists, can identify the threshold and degree of advanced demineralization caries in a tooth, so as to make a sound decision whether and how to treat the lesion locally.

## Data Availability

Data and code developed in this study are available upon reasonable request to the corresponding author.

## References

[1] Balageas D. L. Krapez J.-C. Cielo P., “Pulsed photothermal modeling of layered materials,” J. Appl. Phys. 59(2), 348–357 (1986). JAPIAU 0021-8979 10.1063/1.336690

[2] Long F. H. Anderson R. R. Deutsch T. F., “Pulsed photothermal radiometry for depth profiling of layered media,” Appl. Phys. Lett. 51(25), 2076–2078 (1987). APPLAB 0003-6951 10.1063/1.98985

[3] Schmitt J. M. et al., “Multilayer model of photon diffusion in skin,” J. Opt. Soc. Am. A 7(11), 2141–2153 (1990). JOAOD6 0740-3232 10.1364/JOSAA.7.002141 2254803

[4] Prahl S. A. et al., “Determination of optical properties of turbid media using pulsed photothermal radiometry,” Phys. Med. Biol. 37(6), 1203 (1992). PHMBA7 0031-9155 10.1088/0031-9155/37/6/001 1626021

[5] Vitkin I. A. Wilson B. C. Anderson R. R., “Analysis of layered scattering materials by pulsed photothermal radiometry: application to photon propagation in tissue,” Appl. Opt. 34(16), 2973–2982 (1995). APOPAI 0003-6935 10.1364/AO.34.002973 21052451

[6] Selwitz R. H. Ismail A. I. Pitts N. B., “Dental caries,” Lancet 369(9555), 51–59 (2007). LANCAO 0140-6736 10.1016/S0140-6736(07)60031-2 17208642

[7] Kidd E. Fejerskov O., Eds., “How does a caries lesion develop?,” in Essentials of Dental Caries, Oxford University Press (1998).

[8] Darling C. L. Huynh G. Fried D., “Light scattering properties of natural and artificially demineralized dental enamel at 1310 nm,” J. Biomed. Opt. 11(3), 034023 (2006). JBOPFO 1083-3668 10.1117/1.2204603 16822072

[9] Fried D. et al., “Nature of light scattering in dental enamel and dentin at visible and near-infrared wavelengths,” Appl. Opt. 34(7), 1278–1285 (1995). APOPAI 0003-6935 10.1364/AO.34.001278 21037659

[10] Zuerlein M. J. Fried D. Featherstone J. D., “Modeling the modification depth of carbon dioxide laser-treated dental enamel,” Lasers Surg. Med. 25(4), 335–347 (1999). LSMEDI 0196-8092 10.1002/(SICI)1096-9101(1999)25:4<335::AID-LSM8>3.0.CO;2-F 10534750

[11] Nicolaides L. et al., “Quantitative dental measurements by use of simultaneous frequency-domain laser infrared photothermal radiometry and luminescence,” Appl. Opt. 41(4), 768–777 (2002). APOPAI 0003-6935 10.1364/AO.41.000768 11993925

[12] Matvienko A. et al., “Theoretical analysis of coupled diffuse-photon-density and thermal-wave field depth profiles photothermally generated in layered turbid dental structures,” J. Appl. Phys. 105(10), 102022 (2009). JAPIAU 0021-8979 10.1063/1.3116128

[13] Hellen A. et al., “Quantitative remineralization evolution kinetics of artificially demineralized human enamel using photothermal radiometry and modulated luminescence,” J. Biophotonics 4(11–12), 788–804 (2011). 10.1002/jbio.201100026 21761572

[r14] Jeon R. J. et al., “*In vitro* detection and quantification of enamel and root caries using infrared photothermal radiometry and modulated luminescence,” J. Biomed. Opt. 13(3), 034025 (2008). JBOPFO 1083-3668 10.1117/1.2942374 18601570

[15] Tabatabaei N. Mandelis A. Amaechi B. T., “Thermophotonic lock-in imaging of early demineralized and carious lesions in human teeth,” J. Biomed. Opt. 16(7), 071402 (2011). JBOPFO 1083-3668 10.1117/1.3564890 21806248

[16] Shokouhi E. B. Mandelis A., “Fourier spectral theory of pulsed photothermal radiometry, signal analysis, and parametric measurements in two-layered media,” J. Appl. Phys. 133(11), 114701 (2023). JAPIAU 0021-8979 10.1063/5.0140890

[17] Hellen A. et al., “Optothermophysical properties of demineralized human dental enamel determined using photothermally generated diffuse photon density and thermal-wave fields,” Appl. Opt. 49(36), 6938–6951 (2010). APOPAI 0003-6935 10.1364/AO.49.006938 21173829

[r18] Craig R. G. Peyton F. A., “Thermal conductivity of tooth structure, dental cements, and amalgam,” J. Dent. Res. 40(3), 411–418 (1961). JDREAF 0022-0345 10.1177/00220345610400030501

[r19] Tabatabaei N. et al., “On the sensitivity of thermophotonic lock-in imaging and polarized Raman spectroscopy to early dental caries diagnosis.,” J. Biomed. Opt. 17(2), 025002 (2012). JBOPFO 1083-3668 10.1117/1.JBO.17.2.025002 22463028

[20] Minesaki Y., “Thermal properties of human teeth and dental cements,” cal 1(84), 10 (1990). 2134829

[21] Matvienko A. et al., “Photothermal detection of incipient dental caries: experiment and modeling,” Proc. SPIE 6759, 67590J (2007). PSISDG 0277-786X 10.1117/12.734987

[22] Horne K. et al., “Photothermal radiometry measurement of thermophysical property change of an ion-irradiated sample,” Mater. Sci. Eng. B 177(2), 164–167 (2012). 10.1016/j.mseb.2011.10.014

[r23] Iserles A., “Numerical recipes in C—the art of scientific computing, by W. H. Press, B. P. Flannery, S. A. Teukolsky and W. T. Vetterling. Pp 735. £27·50. 1988. ISBN 0-521-35465-X (Cambridge University Press),” Math. Gaz. 73(464), 167–170 (1989). 10.2307/3619708

[24] Matvienko A. Mandelis A. Abrams S., “Robust multiparameter method of evaluating the optical and thermal properties of a layered tissue structure using photothermal radiometry,” Appl. Opt. 48(17), 3192–3203 (2009). APOPAI 0003-6935 10.1364/AO.48.003192 19516364

[25] Hellen A. et al., “Quantitative evaluation of simulated human enamel caries kinetics using photothermal radiometry and modulated luminescence,” Proc. SPIE 7883, 583–596 (2011). 10.1117/12.878846 21806252

[26] Spitzer D. Ten Bosch J. J., “The absorption and scattering of light in bovine and human dental enamel,” Calcif. Tissue Res. 17, 129–137 (1975). CATRBZ 0008-0594 10.1007/BF02547285 1139366

[27] Spitzer D. Ten Bosch J. J., “Luminescence quantum yields of sound and carious dental enamel,” Calcif. Tissue Res. 24, 249–251 (1977). CATRBZ 0008-0594 10.1007/BF02223324 597765

[28] Braden M., “Heat conduction in normal human teeth,” Arch. Oral Biol. 9(4), 479–486 (1964). AOBIAR 0003-9969 10.1016/0003-9969(64)90033-0 14179056

[29] Brown W. S. Dewey W. A. Jacobs H. R., “Thermal properties of teeth,” J. Dent. Res. 49(4), 752–755 (1970). JDREAF 0022-0345 10.1177/00220345700490040701 5269374

[30] Abou Neel E. A. et al., “Demineralization-remineralization dynamics in teeth and bone,” Int. J. Nanomedicine 11, 4743–4763 (2016). 10.2147/IJN.S107624 27695330 PMC5034904

